# Light Like a Feather: A Fibrous Natural Composite with a Shape Changing from Round to Square

**DOI:** 10.1002/advs.201600360

**Published:** 2016-12-01

**Authors:** Bin Wang, Marc André Meyers

**Affiliations:** ^1^ Materials Science and Engineering ProgramDepartment of Mechanical and Aerospace EngineeringUniversity of CaliforniaSan DiegoLa Jolla, CA92093USA

**Keywords:** feather shaft circular‐to‐square, layered fibrous structures, ovalization

## Abstract

Only seldom are square/rectangular shapes found in nature. One notable exception is the bird feather rachis, which raises the question: why is the proximal base round but the distal end square? Herein, it is uncovered that, given the same area, square cross sections show higher bending rigidity and are superior in maintaining the original shape, whereas circular sections ovalize upon flexing. This circular‐to‐square shape change increases the ability of the flight feathers to resist flexure while minimizes the weight along the shaft length. The walls are themselves a heterogeneous composite with the fiber arrangements adjusted to the local stress requirements: the dorsal and ventral regions are composed of longitudinal and circumferential fibers, while lateral walls consist of crossed fibers. This natural avian design is ready to be reproduced, and it is anticipated that the knowledge gained from this work will inspire new materials and structures for, e.g., manned/unmanned aerial vehicles.

## 1 Introduction

**Figure 1:**
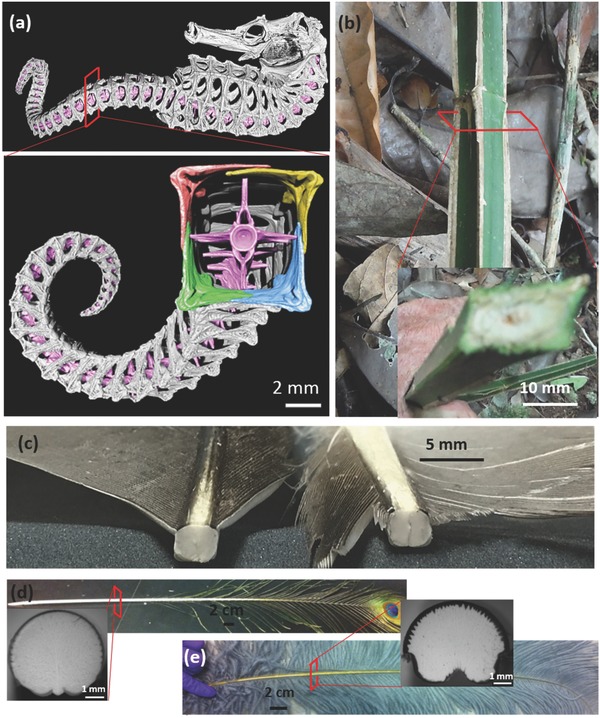
Square shapes in nature: a) seahorse tail skeleton (reproduced with permission.^1^ Copyright 2015, AAAS); b) liana stem from Nambikwara indigenous territory, Amazon; c) cross section of feather rachis from seagull. Cross section of circular rachis from d) peacock tail feather and e) ostrich wing feather.

The square shape in nature has evolved in only a few living organisms. At the structural level, the seahorse tail (**Figure** **1**a) is square and thus more resilient when crushed, preserving its articulatory organization upon bending and twisting.^1^ The Nambikwara liana (Figure 1b) shows a square stem that contains sharp edges and therefore has a protective effect against predators, and possibly increases its stiffness. Within vertebrates, the avian feather rachis also shows a square cross section. At the cellular level, plant cells have rectangular shapes, and porous plant stems have rectangular units. However, these are exceptions to the rule. Although the square seahorse tail has been recently explained,^1^ the square cross section of flight feather rachis, which is distinct from the circular cross section of flightless feather rachis (Figure 1d,e), remains a mystery.

**Figure 2:**
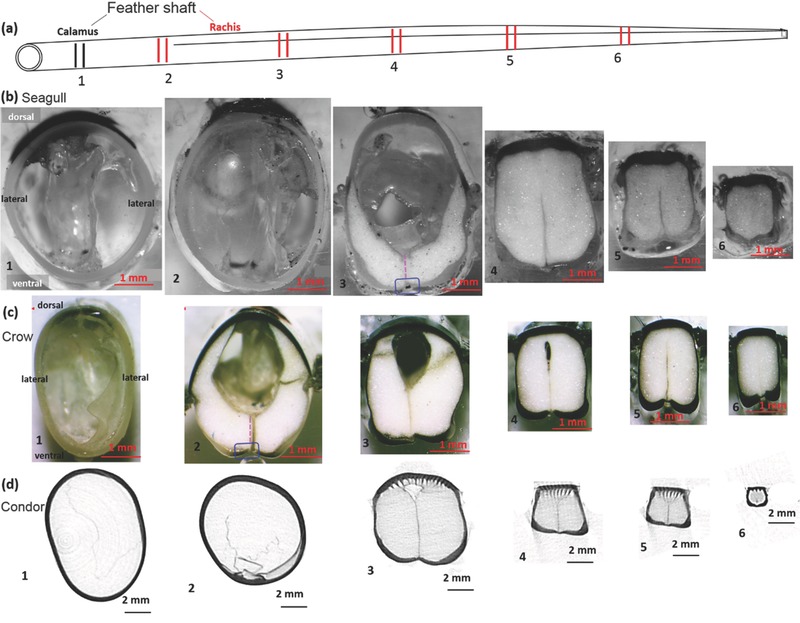
Changing shape along the feather shaft: a) schematic of a flight feather shaft, numbers indicating positions along the shaft length (from calamus to rachis). Optical micrographs of the transverse sections along the shaft from b) seagull and c) crow and microcomputed tomography images from d) condor showing gradual shape change from circular hollow tube to rectangular foam filled. Pink dotted lines indicate the transverse septum and blue rectangles the ventral groove, respectively. Dorsal, lateral, and ventral portions of the shaft cortex are marked in the left figure.

Flight feathers of volant birds, upon encountering aerodynamic forces, aid the generation of thrust and lift, and primarily bend and twist.^2^, ^3^ The central shaft provides the main mechanical support. Feather shafts are lightweight, stiff, and strong, yet sufficiently flexible, properties that have potential for the development of bioinspired materials for both aircraft and structural applications. The inside of the feather shaft is filled with air at the calamus (proximal end) and foam (medulla) at the rachis (middle and distal shaft) (**Figure** **2**).

The feather rachis of different birds has been mainly simplified as a cylindrical shell filled with a foam core, with a focus on cortex properties, such as tensile strength.^4^, ^5^, ^6^, ^7^, ^8^, ^9^ Feathers are based on β‐keratin.^10^, ^11^, ^12^, ^13^, ^14^, ^15^ At the molecular level, keratinization of the feathers occurs by dead keratinocytes whose properties are determined during formation.^16^ This implies that the microstructure is “designed” for intended functions. However, the detailed fibrous structure of the whole shaft is unexpectedly under‐documented. An increase of axially aligned keratin molecules toward the tip within the feather rachis was reported 17; circumferential, axial fibers, and crossed‐fibers were observed by selectively degrading the matrix proteins.^18^ A few studies used nanoindentation to obtain the local modulus and hardness of feathers by indenting in limited or unspecified locations.^2^, ^19^, ^20^

From an engineering perspective, the feather shaft resembles a cantilever beam subjected to distributed loading; both the material properties (Young's modulus, *E*) and the geometry (area moment of inertia, *I*) determine the flexural properties. The latter changes substantially from the proximal to the distal end of the feather shaft,^6^ as the bending moment decreases accordingly. The geometry involves variations not only in size but also in shape.

In a quest to understand the structural design of feather shaft, we explain, for the first time, why its cross sectional shape changes from round to rectangular. Flight feathers from the California Gull (*Larus californicus*) and the American Crow (*Corvus brachyrhynchos*) representing marine and land birds, respectively, were studied.

## 2 Results and Discussion

### 2.1 Shape Factor of the Feather Shaft Cortex

The flight feather shafts from seagull and crow exhibit similar features, shown in Figure 2. The transverse sections of the shaft at the calamus show elliptical compact cortices. In the region between the calamus and the proximal rachis (positions 2 and 3) in Figure 2, the cortex shows a near transitional shape with a groove at the middle of the ventral surface (blue rectangles in Figure 2b,c) where a foamy medulla (substantia medullaris) and a transverse septum (pink dotted lines in Figure 2b,c) start to develop. Toward the distal rachis, the cortex attenuates and the medulla gradually fills the cortex.

**Figure 3:**
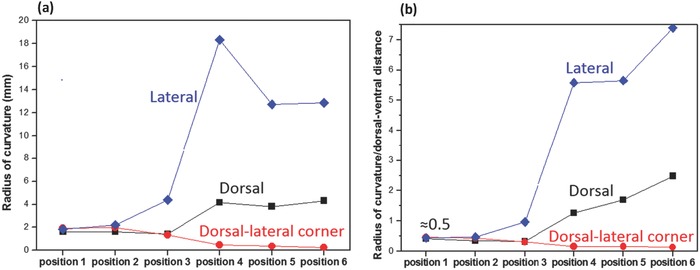
The roundness and squareness along the feather shaft length measured from the seagull primary feather (Figure 2b). a) Measured radii of curvatures of dorsal, dorsal–lateral corner, and lateral regions from the calamus to the distal shaft (represented by positions 1–6). b) Ratios of the radius of curvature of each cortical region over the local dorsal–ventral distance along the shaft length.

A salient feature, the shape change of cortex from circular at the calamus to square/rectangular toward the distal shaft, is strikingly different from that of flightless feathers, e.g., ostrich wing and peacock tail feathers (Figure 1d,e).^21^ These are circular throughout the entire shaft length.^8^, ^20^ Flight feathers from other flying birds, e.g., condor (Figure 2d), pigeon,^6^ barn owl,^2^ pelican, and seriema,^21^ show a similar change in shape factor; this is demonstrated by the squareness along the shaft length, the measured average radii of curvatures of different cortical regions, and the ratios of those radii over the entire cortical size, as plotted in **Figure** **3**. At the calamus (positions 1 and 2), the dorsal, dorsal–lateral corner, and lateral regions exhibit comparable radius of curvature; ratios of each radius of curvature over the local dorsal–ventral distance are all close to 0.5, both indicating the circular cross sectional shape. Towards the distal shaft, the dorsal and lateral regions show clearly increasing radius of curvature and increasing ratio of the radius of curvature over the local dorsal–ventral distance; while the dorsal–lateral corner shows obviously a decrease in radius of curvature and decrease in ratio of the radius of curvature over the dorsal–ventral distance. These evidence the shape change from circle/ellipsis to rectangle. The dorsal region shows to a smaller degree the increase in the radius of curvature, which is due to its convex shape.

In addition, toward the distal rachis, the dorsal and ventral cortices are much thicker than the lateral walls (approximately ten times thicker). This resembles a human‐made I‐beam where the majority of material is distributed at the upper and lower regions to resist the maximum stresses. Interestingly, the rectangular/square cortex at the distal rachis shows a slightly shorter height on one lateral wall (facing front, the leading edge 22).

We show here that the shape change of cortex from round to rectangular plays a pivotal role in adjusting the area moment of inertia and thus the flexural rigidity along the shaft length. The shaft tapers toward the distal end, thus minimizing the deflection/weight ratio by tailoring the amount of material, shape and dimensions along the shaft. It does this by modulating the bending rigidity (product of *E* and *I*), to sustain the complex forces at the base and to minimize the increasing deflection toward the distal rachis. The area moment of inertia, *I*, is correlated with the amount of material and the cross sectional shape of a beam; a uniformly high value of *I* using a large amount of material would be mechanically favorable but would produce a weight penalty.^4^, ^17^ It will be shown below that changing the shape is an ingenious solution to enhance the bending rigidity while decreasing the overall weight of the feather.

### 2.2 Flexural Advantages of Square Tubes over Circular Ones

Beams with the same cross sectional area but different shapes give different area moments of inertia, e.g., for circular and square beams with the same cross sectional area (*a* ^2^ =  *πr* ^2^), the square one has larger *I* $(I_{\mathrm{squ}}=\frac{a^{4}}{12}=\frac{\pi r^{4}}{3.8}$ > $I_{\mathrm{cir}}=\frac{\pi r^{4}}{4})$ and thus higher flexural rigidity. Importantly, a square cross section has advantages over a circular one in resisting cross sectional shape change during bending. The calamus needs to be circular to penetrate smoothly into and connect efficiently with the tissue; once coming out of skin, the rachis gradually becomes rectangular after ≈20% shaft length. The flexural behavior of hollow tubes, which feather shafts resemble, involves both the shape and the material's structure.^23^

#### 2.2.1 Flexural Behavior

**Figure 4:**
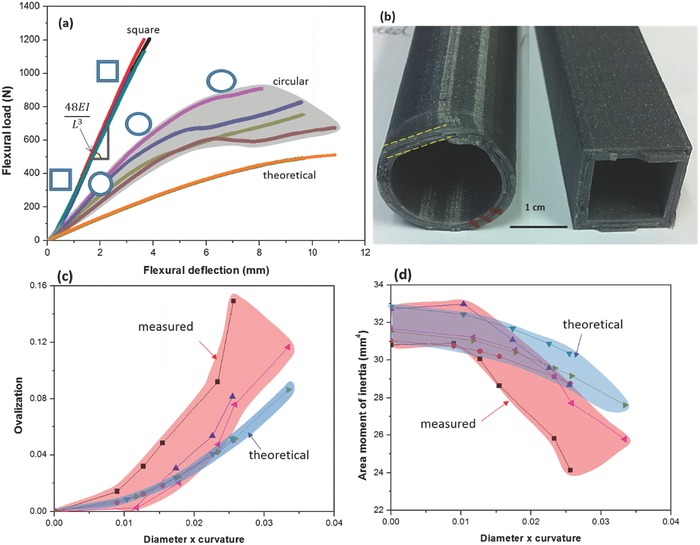
Flexural behavior of hollow tubes. Square tubes show linear flexure load versus deflection response, whereas circular ones show curves with lower slope (flexural rigidity over *L^3^*) and undergo ovalization of cross section with increasing loading, leading to a decrease in area moment of inertia and corresponding decrease in rigidity. a) Flexural load–deflection curves of the 3D‐printed PLA tubes with circular and square sections, overlaid with theoretical calculated curves of circular tubes considering ovalization; b) photograph of the fractured surfaces of circular and square PLA tubes. c) Measured degree of ovalization versus bending curvature (dimensionless) for PP hollow cylindrical tubes in pure bending, and d) measured area moments of inertia versus bending curvature (dimensionless); overlaid plots in blue are from theoretical calculations.

We examine the bending response of 3D printed PLA (polylactic acid, polymer) tubes with the same cross sectional area to answer the question: why does the feather shaft choose a square shape toward the distal rachis? The flexural load–deflection curves of all tubes are plotted in **Figure** **4**a. Square tubes show consistently higher slope, and the flexural rigidity and modulus (Supporting Information, Section I) are ≈24.2% larger than circular ones. This indicates the higher efficiency (higher ability per unit area) of square tubes (representing the rachis) in resisting bending and minimizing flexural deformation than the circular ones that represent the calamus. In addition, circular tubes exhibit load–deflection responses that deviate significantly from the initial linear region, indicating a decreasing value of *I* due to cross sectional shape change from circular to oval. This effect is called “ovalization”. Figure 4b shows that the circular tube exhibits a certain degree of ovalization (dashed lines); whereas the cross section of square tube retains almost the original shape.

The square tube delays the onset of shape change because of flat and large contact area that relieves stress concentration, whereas the circular tube readily undergoes ovalization due to loading on a much smaller contact region. In addition, the orthogonal edges of square tubes restrict further transverse deformation and thus resist the cross‐sectional shape change. For circular tubes, the flattening/ovalization initiates at the loading point, gradually invading the entire cross section, leaving less material in the original shape (circular) to sustain load. The larger the cross‐sectional shape change, the greater the decrease in *I*, and the less the ability to resist further flexural force.

This also indicates that the changing cross‐sectional shape to square, which provides higher flexural rigidity, can partially counterbalance the large reduction in *I* caused by the tapering of the shaft toward the distal free end to reduce profile drag,^4^, ^24^ save energy, and decrease the weight. This effect is demonstrated analytically below.

#### 2.2.2 Pure Bending

The ovalization of circular tubes, called Brazier effect^25^ (degree of ovalization, ζ, Supporting Information, Section II), affects the bending rigidity. We present the change in area moment of inertia as a function of increasing bending moment and compare it with experimental results on polypropylene (PP) tubes of various diameters (7.4–11.5 mm). At a given bending curvature, the measured degree of ovalization in the cylindrical tube is

(1) $$\zeta_{\mathrm{me}}=\frac{d-b}{d}$$

where *d* is the measured original diameter and *b* is the measured minor axis of the ovalized cross section (vertical height) of the tube. The corresponding area moment of inertia is

(2) $$I_{\mathrm{me}}=\frac{\pi }{4}\left(\frac{a}{2}\right)\;{\left(\frac{b}{2}\right)}^{3}-\frac{\pi }{4}\left(\frac{a}{2}-t\right)\;{\left(\frac{b}{2}-t\right)}^{3}\approx \frac{\pi }{4}\frac{\left(b^{3}+3b^{2}a\right)}{8}t$$

where *a* is the measured major axis of the ovalized cross section (horizontal dimension) of the tube. Figure 4c shows plots of ζ_me_ versus bending curvature (=diameter × curvature) of representative tubes. With increasing bending curvature, tubes show an increasing degree of ovalization (Figure 4c), and an associated decreasing area moment of inertia (Figure 4d).

This ovalization can also be theoretically calculated; upon bending, ovalization minimizes the total strain energy of the system. Thus a theoretical degree of ovalization is^26^

(3) $$\zeta_{\mathrm{th}}=\kappa^{2}r^{4}\frac{\left(1-\nu^{2}\right)}{t^{2}}$$

where κ, *r*, and *t* are the bending curvature, original radius, and thickness of the tube, respectively, and ν is Poisson's ratio of the material. The theoretical area moment of inertia can be obtained as a function of the degree of ovalization^26^

(4) $$I_{\mathrm{th}}=\pi r^{3}t\left(1-\frac{3}{2}\zeta_{\mathrm{th}}+\frac{5}{8}\zeta_{\mathrm{th}}^{2}\right)$$

where ζ_th_ is calculated from Equation (3). The theoretically calculated degree of ovalization and area moment of inertia as a function of bending curvature are overlaid on experimentally measured values in Figure 4c,d.

There is good agreement. The calculated degree of ovalization of all types of tubes, with increasing bending curvature, increases monotonically, and agrees with the experimentally measured values. For the area moment of inertia, both theoretical and experimental values decrease with increasing bending curvature. The theoretical area moment of inertia versus bending curvature closely reflects the experimental results. The measurements and calculations demonstrate the intrinsic deficiency of a circular tube in maintaining the original area moment of inertia, thus deteriorating the flexural rigidity.

This theoretical ovalization can be used to determine theoretical flexural load–deflection curves for the circular PLA tubes in three point bending. An expression for the bending curvature as a function of the deflection at the center point is derived as (Supporting Information, Section III)

(5) $$\kappa =\frac{16\delta }{L^{2}}$$

For each measured δ (deflection), using Equations (3), (4) and (5), we obtain the theoretical area moment of inertia *I*_th, δ_; substituting this expression into the equation for a center‐loaded beam (Equation (S6) in the Supporting Information, Section III), the flexural load is calculated. The plot is labeled “theoretical” and presented in Figure 4a; the curves are below the measured values for circular tubes but show the same trend as experiments. These calculations demonstrate that the square hollow tube provides a greater rigidity, normalized per weight, than the circular one.

### 2.3 The Layered Fibrous Structure of Cortex

The second focus of this contribution is to reveal and understand the changing arrangement of the fibrous keratin in the father shaft, and to augment the knowledge from previous reports.^[3,5,17]^ The feather cortex can be considered as a fiber‐reinforced composite: at the nanoscale, it consists of crystalline β‐keratin filaments (≈3 nm in diameter) embedded in amorphous matrix proteins^22^; both compose macrofibrils (≈200 nm in diameter), which are surrounded by amorphous inter‐macrofibrillar material. These two components further organize into fibers (3–5 µm in diameter) which are often seen in fractured rachis under a scanning electron microscope (Figure S3, Supporting Information).

**Figure 5:**
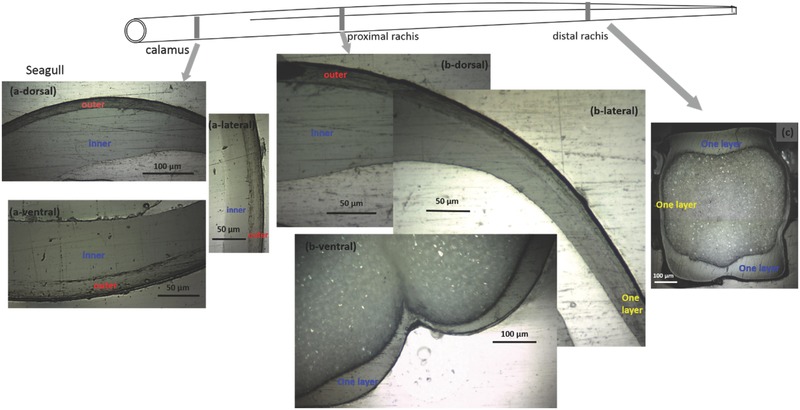
Change in keratin fiber orientation along shaft length (seagull). Transverse sections of shaft along shaft length showing the layered structure from seagull. Note central figures not in proportion. a) At the calamus, the dorsal, lateral walls, and ventral regions all clearly show a thin outer layer and a thick inner layer. b) At the proximal rachis, the outer layer exists in dorsal region but becomes thinner and disappears in lateral wall, and only one layer is present in ventral region. c) At the distal rachis, the entire cortex, including dorsal, ventral, and lateral walls, shows only one layer.

The cortices of both seagull and crow feathers show a complex layered structure composed of differently oriented fibers along the shaft length, which correlates to the mechanical functions changing from the calamus to the distal rachis. Sectioning and polishing reveal the layers. At the calamus, the entire cortex (dorsal, lateral walls, and ventral regions) of seagull feather consists of a thin outer layer and a thick inner layer. At the proximal rachis, the dorsal region of cortex shows a thinner outer layer and a thick inner layer, but toward the lateral walls the outer layer gradually disappears with only one layer present (**Figure** **5**b‐lateral). The ventral region shows a uniform single layer (Figure 5b‐ventral). At the distal rachis, no outer layer is observed for the entire cortex. The crow feather shows similar features, seen in Figure S4 of the Supporting Information.

**Figure 6:**
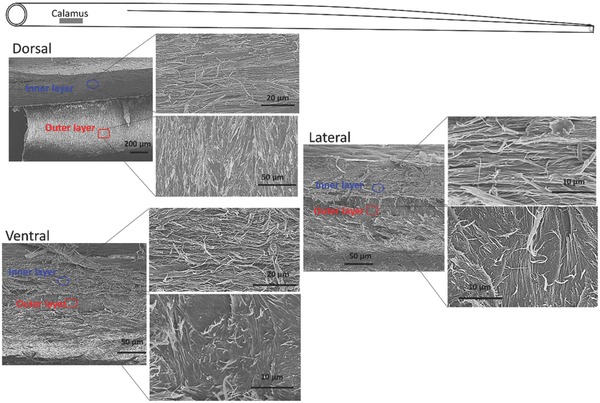
The fiber orientations in the cortex at the calamus. Scanning electron micrographs of the longitudinal sections at the different cortical regions from seagull feather: the dorsal, lateral, and ventral regions all show a thick inner layer formed by axial fibers and an outer layer of circumferential fibers (the view is looking from the internal surface of the cortex).

**Figure 7:**
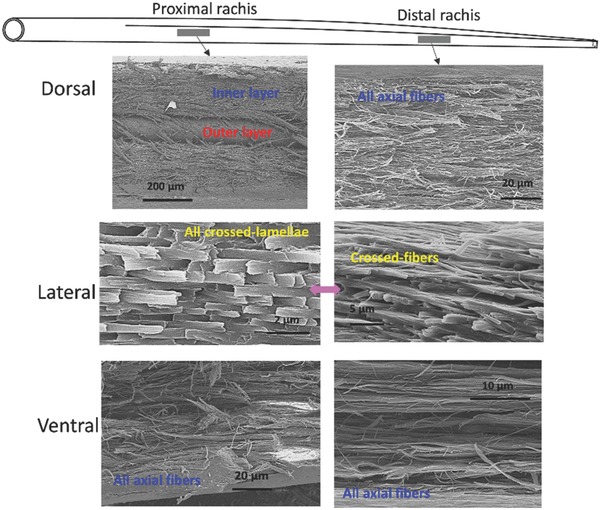
The fiber orientations in the cortex at the proximal and distal rachis. Scanning electron micrographs of the longitudinal sections at different cortical regions: at the proximal rachis, the dorsal region shows an inner layer of axial fibers and an outer layer of circumferential fibers, whereas the lateral walls show crossed‐lamellae and the ventral region exhibits only axial fibers. At distal rachis, both the dorsal and the ventral region are composed of axial fibers, and the lateral walls of crossed‐lamellae. The crossed lamellae are indicative of a crossed‐fiber structure.

Fracture of the feather cortex along the dorsal, lateral, and ventral longitudinal sections reveals the orientations of the aligned fibers along shaft length (**Figures** **6** and **7**). At the calamus, the entire cortex exhibits a thick inner layer composed of longitudinally (axially) oriented fibers and an outer layer of sheets of circumferentially aligned fibers, shown in Figure 6. These layers restrain the axial fibers from separating and prevent axial splitting in flexure. Interestingly, it is a strategy commonly used in the design of synthetic composites. At the proximal rachis, the dorsal cortex shows a thick inner layer of axial fibers covered by circumferential fibers, which are at an obtuse angle to the shaft axis; the ventral cortex is composed of solely axial fibers. The lateral walls, made visible by freeze‐fracture, consist of crossed‐lamellae (Figure 7). At the distal rachis, where only one layer is present in the cortex, the dorsal and ventral regions are all composed of axial fibers while the lateral walls consist of crossed‐lamellae, which are indicative of crossed‐fibers (Figure 7). The crow feather shaft cortex shows the same fibrous structure (Figure S5, Supporting Information). This cross‐lamellar structure is important in tailoring the lateral rigidity, which is much lower than the dorsal‐ventral rigidity. The fibers, being at angle to the longitudinal axis, can flex in compression and slide in tension, thus creating a desirable decrease in lateral rigidity. This is yet another fascinating aspect of the anisotropic rigidity of the flight feathers.

The increase in axial fibers and decrease in circumferential fibers are important to the flexural properties of feather shaft. The flexural rigidity is the product of *I* and the longitudinal Young's modulus (*E*).^27^ The latter is determined by the local fibrous structure. As the shaft bends, the cortex at calamus (circumferential fibers enclosing axial fibers) provides robust mechanical support. Cameron et al.^17^ reported a higher value of *E* toward the rachis tip due to a higher proportion of axially aligned fibers. Here we confirm that in the dorsal and ventral cortices the amount of axially aligned fibers increases, which leads to a higher *E* of rachis toward the distal end; thus, again, compensating for the decrease in *I* due to the reduced material to ensure necessary flexural rigidity.

The entire lateral cortex of both rachises consists of crossed‐lamellae, which are formed by crossed fibers (directly observed); they can provide necessary dorsal–ventral flexibility and prevent damage to the feather shaft. During bending, the dorsal and ventral cortex provide stiffness, while the lateral walls allow the shaft to flex with desirable strain under loading, thus delaying the onset of buckling and failure.^18^ Besides, the crossed‐fibers structure may be a key in limiting damage from barbs. The barbs, carrying arrays of hooked barbules, anchor to the rachis at the lateral walls and generate larger displacements 28, 29 and multi‐directional stresses. A crossed‐fiber structure is more robust in sustaining the displacements and resists the stresses better than axial fibers, which are anisotropic and would be prone to split.

Additionally, the crossed‐fibers can enhance the torsional rigidity, thus controlling twisting during lift or strike. The crossed‐fibers are aligned 45° to the shaft axis, the same orientation to the largest stress in which the material will fracture/split under torsion.^30^ At the same time, this torsional rigidity is complemented by the axial fibers in the dorsal and ventral cortex and the cortex shape, which facilitate twisting. Twisting lowers the bending moment before causing local buckling of thin‐walled cylinders^5^, ^31^ and dissipates energy to avoid permanent damage. Therefore, the crossed‐fibers in the lateral walls and the predominant axial fibers covered by a gradual decrease of circumferential fibers in the dorsal and ventral cortex work synergistically to provide optimized mechanical functions to the shaft.

As a fibrous composite, the superior mechanical properties of the feather cortex are in the fiber direction.^32^, ^33^ As keratin proteins are cross‐linked intracellularly^34^ and there is no evidence that the filaments pass through the cell membrane complex,^35^ a possible length of a β‐keratin filament and a macrofibril would be the cell length (20–50 µm); therefore, the β‐keratin filaments, macrofibrils and fibers are long compared with their width,^35^ and the mechanical behavior is close to that of a composite with continuous fibers.^35^, ^36^

Nanoindentation was used to interrogate the fibrous structure. There are subtle changes which correlate with the orientations of the keratin fibers along the shaft length; there are also changes from the dorsal to the lateral and ventral regions of cortex, consistent with the observed fiber alignment. The results, shown in the Supporting Information, Section VI, confirm the complex nature of the cortex, where fiber alignment maximizes rigidity and failure resistance.

## 3 Conclusions

**Figure 8:**
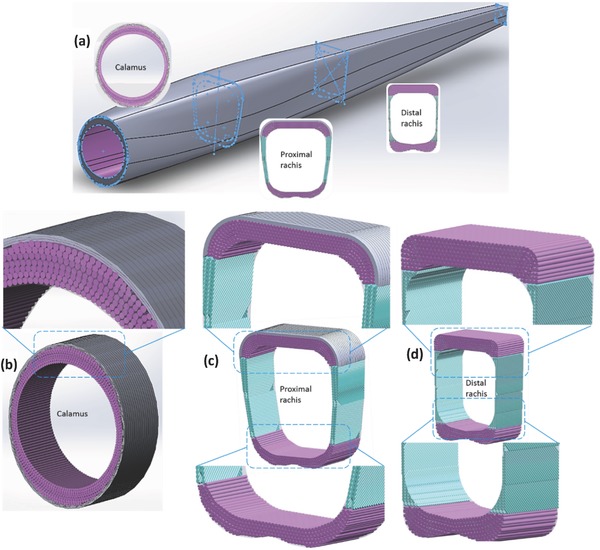
Structural model of the feather shaft cortex: a) the shape factor. The cross section changes from circular at the calamus to near rectangular at the rachis. The layered structure of cortex with varying and differentially oriented fibers along shaft length: b) at the calamus, all the cortex is composed of a thin outer layer of circumferential fibers and a thick inner layer of aixal fibers; c) at the proximal rachis, the dorsal cortex consists of a thinner outer layer of circumferential fibers covering axial fibers, the lateral walls of crossed fibers and the ventral cortex of longitudinal fibers; d) at the distal rachis, the dorsal and ventral cortices are composed of axial fibers and the lateral walls of crossed fibers.

The current findings of the feather shaft cortex involving a cross sectional shape change and a complex layered fibrous structure along the shaft length to fulfill the flight functions are illustrated in schematic fashion in **Figure** **8**:

- *Shape factor*: The cross section of cortex changes from circular at the proximal (calamus) to rectangular toward the end (distal rachis), with significantly thickened dorsal and ventral cortices. This provides higher bending rigidity per unit area and increases the ability to resist sectional shape change during flexure to retain the initial rigidity. The shape also allows twisting under dangerously high loading, thus avoiding failure.
- *Layered fibrous structure*: At the calamus, the entire cortex shows a bulk inner layer of axial fibers covered by a thin (15%) outer layer of circumferential fibers. For the dorsal and ventral cortex, the outer layer becomes thinner as the axially aligned fibers gradually compose the entire dorsal and ventral cortex toward the distal rachis, whereas the lateral walls for the entire rachis show a crossed‐fiber structure (Figure 8b).
- *Synergy*: The shape factor and fibrous morphology create a structure that is longitudinally strong, dorsal‐ventrally stiff, and torsionally rigid, yet capable of prescribed deflection and twisting at a minimum of weight, modulated along the shaft length.

Such a natural design is ready to be reproduced, e.g., using 3D printing or composite manufacturing techniques, and has potential engineering impact in applications such as manned or unmanned aerial vehicles.

## 4 Experimental Section

*Materials*: Flight feather shafts from a California gull (juvenile) and an American crow were used for structural analysis and mechanical testing. The feathers were obtained after the natural death of the birds and stored and studied at room temperature and humidity.

*Structural Characterization*: For optical microscopy, the feather shafts were cut into small cylindrical parts at different positions along shaft axis from proximal to distal end (numbered as 1, 2, 3, 4, 5, and 6), embedded in epoxy with transverse and longitudinal sections exposed, and polished using graded sand papers up to 2400# and finally polishing paste (0.3 µm aluminum oxides). For microcomputed tomography scan, transverse sections along the feather shaft were scanned with a scanner (Skyscan 1076, Kontich, Belgium) at 36 µm isotropic voxel sizes. Images were developed using Skyscan's DataViewer and CTVox software. For scanning electron microscopy, transverse and longitudinal sections of feather shaft segments were obtained by cutting and folding or breaking at different positions along the shaft length, and then coated with iridium for observation. The lateral walls of feather rachis cortex were submerged in liquid nitrogen, manually fractured in longitudinal direction and coated with iridium. An Axio Fluorescence microscope and a Phillips XL30 environmental scanning electron microscope at Nano3 facility at Calit2, UCSD, were used.

*Nanoindentation*: The feathers shafts cut into six cortex segments of ≈4 mm in height from proximal (calamus) to the distal end (feather tip). The segments were numbered as 1, 2, 3, 4, 5, and 6 representing their normalized distance from feather proximal point (see Figure 2a in main text). They were mounted in epoxy and the transverse sections were polished in the same way as for structural observation (graded sand papers and 0.3 µm polishing paste). Then the mechanical variation of dorsal, lateral, and ventral regions along shaft length (#1→#6) was investigated via indenting on transverse sections of the six cortex sections. In addition, mechanical variation along dorsal cortex thickness on transverse sections at positions #2 and #6 (representing the calamus and the distal rachis) was examined via indenting on dorsal cortex;

All specimens were placed in a fume hood for 2 d and stored in dry containers prior to testing. The specimens were fixed on a steel block using Super Glue and care was taken to ensure that the glue layer was thin enough to have minimal impact on material testing procedures. A nanoindentation testing machine (Nano Hardness Tester, Nanovea, CA, USA) and a Berkovich diamond tip (Poisson's ratio of 0.07 and elastic modulus of 1140 GPa) were used. All specimens were indented with 20 mN of maximum force, a loading and unloading rate of 40 mN min^−1^, and 20 s of creep.

The hardness and reduced Young's moduli were calculated from the load–displacement curves according to ASTM E2546 and the Oliver Pharr method,^37^, ^38^ which is installed in the Nanovea tester (Supporting Information, Section VII). A value of 0.3 for Poisson's ratio of feather keratin was used according to the reported values of keratinous materials in the literature (0.25 for sheep horn^39^; 0.3 for fingernails^40^; 0.37–0.48 for hair keratin^41^). An average of five consistent measurements for each position was reported.

*Pure Bending of Circular Tubes*: Three types of polymeric tubes (thin circular hollow straws) with different diameters and thicknesses were used. The elastic moduli of the straw materials were determined by cutting dog‐bone shape pieces along the axis of the straws, gluing the ends in sand paper, and stretching the specimens in an Instron machine (Instron 3343). All straw materials have similar elastic modulus (≈1 GPa). The two ends of each tube were inserted by fitted tapered inserts, and the loads were applied downward onto the two distal ends, creating a uniform bending moment within the central region (Figure S1, Supporting Information). A camera captures images during bending to measure the height and width of the arc (bent tube); thus the bending radius is calculated to obtain the curvature (*k*). A digital caliper measures the dimensions of the cross section at the middle of the tube as loading increases (horizontal and vertical distances corresponding to major and minor axes of the ovalized cross section), so that the measured degree of ovalization (defined as ζ = δ/*r*; Figure S1, Supporting Information) can be obtained. At least three tubes for each type were tested and measured.

*Three‐Point Bending*: 3D printed polymer tubes (PLA) with square and circular cross sections were used to study the underlying mechanical principles of the shape factor by flexural test. Both types of PLA tubes have the same wall thickness (2.54 mm) and cross sectional area. The external dimensions were 21 and 25 mm for square and circular tubes, respectively; the tube length was 203 mm. Figure 4 shows the flexural load versus deflection curves and a photograph of the PLA tubes, and the span length is 5.8 times the specimen depth. An Instron 3367 equipped with 30 kN load cell was used, and all specimens were tested at room temperature at a nominal strain rate of 10^−3^ s^−1^. Calculations and plots were done using Excel and Origin 8.5.

## Supporting information

As a service to our authors and readers, this journal provides supporting information supplied by the authors. Such materials are peer reviewed and may be re‐organized for online delivery, but are not copy‐edited or typeset. Technical support issues arising from supporting information (other than missing files) should be addressed to the authors.

Supplementary
