# Supplementary material for: Light Like a Feather: A Fibrous Natural Composite with a Shape Changing from Round to Square
Source: Adv Sci (Weinh). 2016 Dec 1;4(3):1600360. doi: 10.1002/advs.201600360 (PMC5357985; doi:10.1002/advs.201600360)
Supplement: Supplementary file 1 — Supplementary [file ADVS-4-na-s001.pdf]

Copyright WILEY-VCH Verlag GmbH & Co. KGaA, 69469 Weinheim, Germany, 2016.

ADVANCED SCIENCE
Open Access

Supporting Information

for *Adv. Sci.,* DOI: 10.1002/advs.201600360

Light Like a Feather: A Fibrous Natural Composite with a Shape Changing from Round to Square

*Bin Wang and Marc André Meyers**

Supporting Information:

I. Three-point bending on hollow square and circular PLA tubes.

For three-point bending, the flexural rigidity is calculated as [1]:

$$EI = \frac{dF}{d\delta}\frac{L^3}{48}$$

(S1)

where E is the flexural modulus, F, δ and L are the flexural load, flexural deflection and supporting span. The flexural modulus is thus:

$$K = \frac{EI}{I}$$

(S2)

where I is the initial area moment of inertia of the tubes ($I_{circular} = \frac{\pi[r^4-(r-t_c)^4]}{4}$, where r and t_c are the radius and thickness; $I_{square} = \frac{a^4-(a-2t_s)^4}{12}$, where a and t_s are the side length and thickness).

II. Ovalization and pure bending of thin polymeric circular tubes.

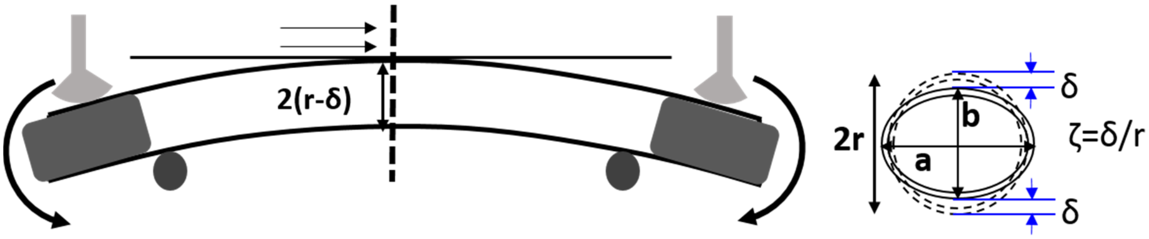

Figure S1. Pure bending of straws and the Brazier effect: uniform bending moment is applied by loading on the two ends of the thin hollow circular tubes, and the original circular cross section (dashed circles) at the middle of the tube deforms into an oval shape. The degree of ovalization, ζ, is characterized by the ratio of δ over r.

III. Theoretical derivation of bending curvature as a function of deflection for three-point bending.

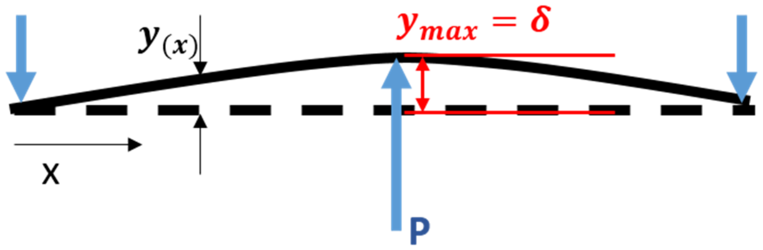

Figure S2. Free body diagram of a simply loaded beam with concentrated load at center.

From a simply supported beam (length L) with concentrated load (P) at center, as shown in Figure S2, the vertical deflection, y(x) is:

$$y_{(x)} = \frac{Px}{48EI}(3L^2 - 4x^2) \qquad (0 \le x \le \frac{L}{2})$$

(S3)

We assume flexural rigidity (*EI*) constant, then the bending curvature is:

$$\kappa = \frac{1}{\rho_{(x)}} = y'' = \frac{d^2y}{dx^2} = -\frac{Px}{2EI}$$

(S4)

At *x*=*L/2*, $P = \delta\frac{48EI}{L^3}$. Plugging these two into Eq. (S4), we obtain the bending curvature at the center of the beam as:

$$\kappa = \frac{16\delta}{L^2}$$

(S5)

For each measured δ, we can calculate the curvature; then, using the theory of ovalization (Eqns. (3), (4) in main text), we obtain the degree of ovalization and corresponding area moment of inertia, $I_{th,\delta}$. At the center of beam, the load is related to deflection δ by,

$$P = \frac{48E}{L^3}\delta I_{th,\delta}$$

(S6)

We can calculate the force for each measured deflection δ, and therefore obtain the theoretical flexural load-deflection curves incorporating ovalization.

IV. Fibers at micrometer scale in the feather shaft cortex usually observed under a scanning electron microscope.

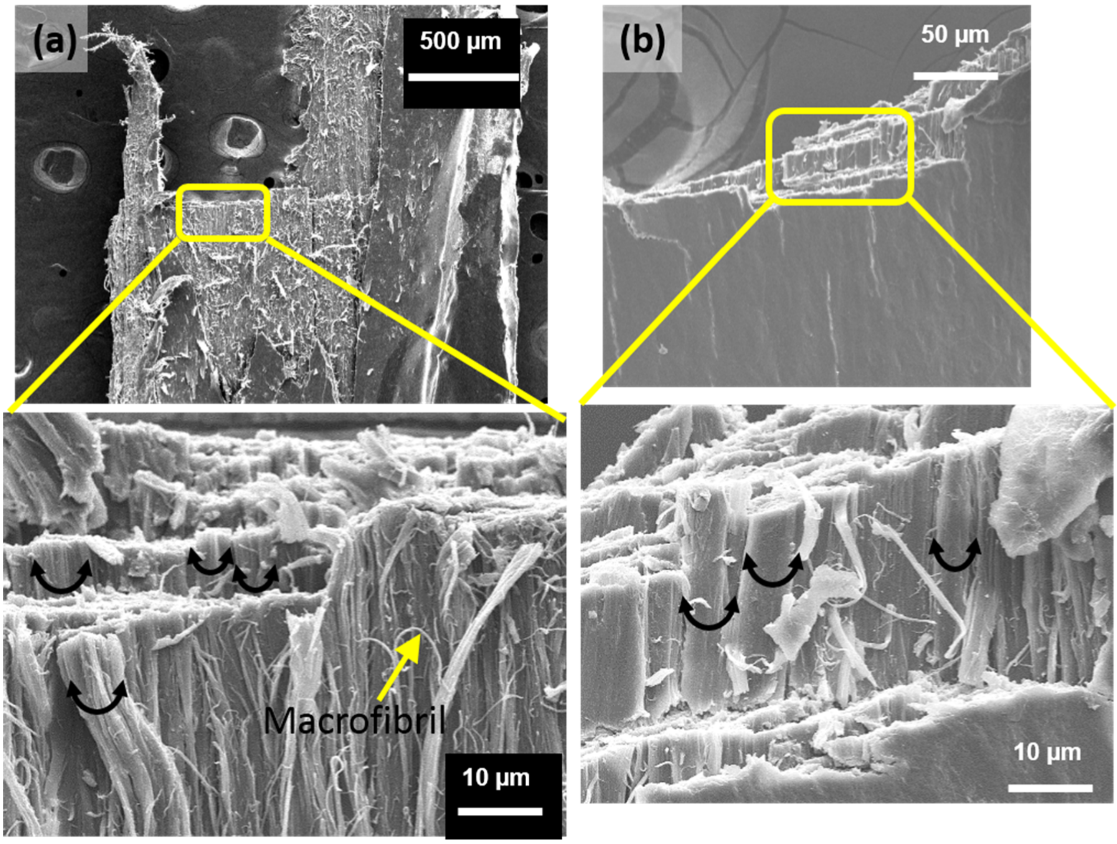

Figure S3. Scanning electron micrographs of the fibers measuring about 3~5 µm (double headed arrows) from (a) crow and (b) seagull feathers. Macrofibrils with diameter (100~200 nm) can also be seen.

V. The layered fibrous structure of crow feather shaft.

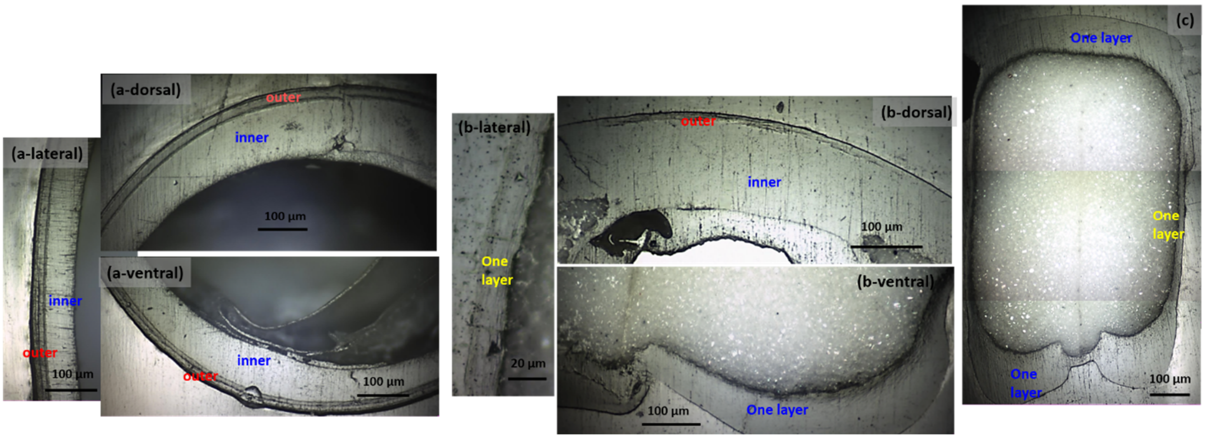

Figure S4. Transverse sections along the shaft length showing the layered structure from crow. (a) At calamus, the dorsal, lateral wall and ventral regions all clearly show a thin outer layer and a thick inner layer. (b) At proximal rachis, the outer layer exists in dorsal region but becomes thinner and disappears in lateral wall, and only one layer is present in ventral region. (c) At distal rachis, the entire cortex, including dorsal, ventral and lateral walls, shows only one layer.

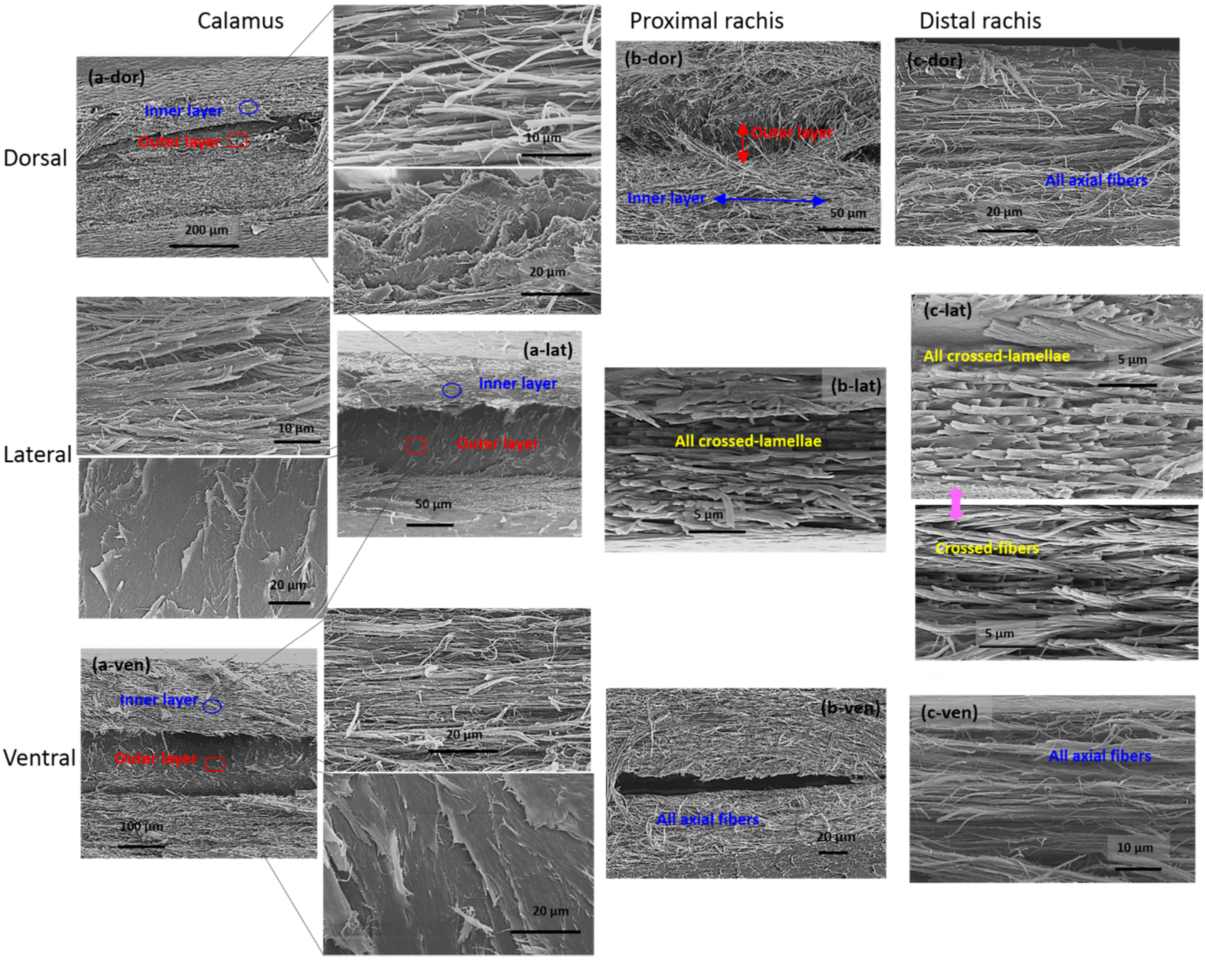

Figure S5. Longitudinal sections at the dorsal, lateral and ventral regions of the cortex from crow feather: at calamus, the (a-dor) dorsal, (a-later) lateral wall, (a-ven) ventral regions all show the thick inner layer formed by axial fibers and the outer layer by circumferential fibers (the view is looking from the internal surface of the cortex). At proximal rachis, (b-dor) the dorsal region shows the inner layer of axial fibers and the outer layer of circumferential fibers, whereas (b-lat) the lateral walls show crossed-lamellae and (b-ven) the ventral region exhibits only axial fibers. At distal rachis, both (c-dor) the dorsal and (c-ven) the ventral regions are composed of axial fibers, and (c-lat) the lateral walls of crossed-lamellae. The crossed-lamellae are indicative of crossed-fibers structure.

VI. Nanoindentation results and analysis

The differences in nanoindentation modulus and hardness on the feather cortex are the results of changes in fiber orientation. In the simple case of a composite with uniaxially aligned fibers, the modulus with loading parallel to the fiber orientation is higher than that with loading perpendicular to them: loading parallel to fibers will allow the force endured by fibers and place them under compression (Figure S6a). Fiber buckling is impeded by the surrounding amorphous phase. When loading is applied, it tends to separate the fibers and a portion of the force goes into the softer matrix (Figure S6b). Nanoidentation measurements made on the transverse section along dorsal cortex at the calamus (#2) and the distal rachis (#6) are shown in Figure S6c,d for seagull and S6e,f for crow. At the calamus, the decrease in modulus and hardness from the inner layer to the outer layer indicates the change of fiber orientations (from indenting on longitudinal fibers to circumferential fibers). For seagull and crow, the modulus is ~7.5 GPa and decreases to ~5.0 GPa in the outer layer. In the distal rachis, the outer layer of circumferential fibers no longer exists and only longitudinally arranged fibers form the dorsal and ventral cortex. Correspondingly, the hardness and modulus are uniform across the entire normalized distance.

The local mechanical properties of lateral walls also exhibit different variation from that of the dorsal and ventral cortex along the shaft length (Figure S6g,h). The hardness of the lateral wall shows a decrease from the proximal region (calamus) to the distal end (distal rachis) of the feather shaft because the crossed-fiber structure starts in the rachis and is not present in the calamus. On the other hand, the dorsal and ventral regions of the feather cortex show only a slight variation or constancy throughout the length (from proximal to distal).

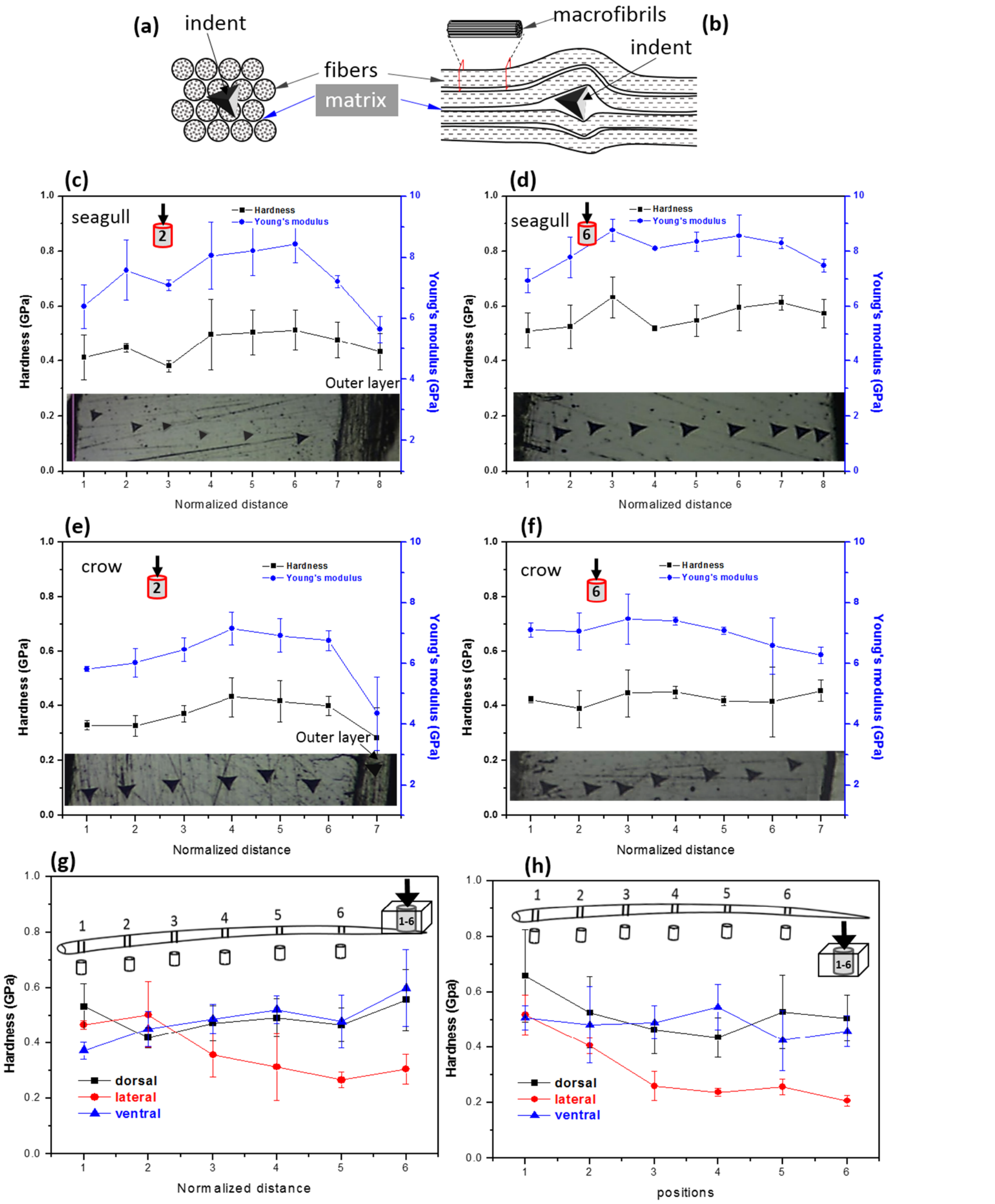

Figure S6. Schematic of indenting on a composite with uniaxial fibers and nanoindentation results: (a) indenting parallel and (b) perpendicular to the fiber directions. The fibers are composed of macrofibrils in the feather cortex. Nanoindentation results along dorsal cortex thickness from inner to outside: (c) at calamus (position #2) and (d) distal rachis (position #6) for seagull, and (e) at calamus (position #2) and (f) distal rachis (position #6) for crow. Nanoindentation results of dorsal, lateral and ventral regions along

the shaft length for (a) seagull and (b) crow. Each data point represent average of five measurements and error bars represent standard deviations.

VII. Calculation of the reduced Young's modulus and hardness from nanoindentation.

The initial unloading curve (90~50% data) is fitted by a power law, and the contact stiffness, *S*, is given by the slope of this line:

$$S = \frac{dP}{dH}$$

(S7)

where *P* is the indentation load and *H* the indentation depth. The reduced Young's modulus E_r is defined as

$$E_r = \frac{\sqrt{\pi}}{2\beta} \cdot \frac{S}{\sqrt{A_c}}$$

(S8)

where is β a constant depending on geometry of the indenter (1.034 for the Berkovich indenter), and A_c is the projected contact area calculated at a depth of indentation, *H*. The Young's modulus of the specimen, E_s, can be obtained from Eqn. (S9):

$$\frac{1}{E_r} = \frac{1-\nu_s^2}{E_s} + \frac{1-\nu_i^2}{E_i}$$

(S9)

where E_i and ν_i are the Young's modulus and Poisson's ratio of the indenter, and ν_s the Poisson's ratio of specimen. A value of 0.3 for Poisson's ratio of feather keratin was used according to the reported values of keratinous materials in the literature (0.25 for sheep horn [2]; 0.3 for fingernails [3]; 0.37-0.48 for hair keratin [4]. The hardness is determined by Eqn. (S10):

$$H = \frac{P_{max}}{A_c}$$

(S10)

where P_{max} and A_c are the maximum load and the projected contact area. An average of five consistent measurements for each position and orientation was used for analysis.

References

[1] R. W. Fitzgerald, *Mechanics of Materials*, Addison-Wesley Publishing Company, Inc., **1982**.

[2] F. L. Warburton, *J. Text. Inst. Proc.* **1948**, *39*, 297.

[3] L. Farran, A. R. Ennos, M. Starkie, S. J. Eichhorn, *J. Biomech.* **2009**, *42*, 1230.

[4] J. Lee, H. J. Kwon, *Int. J. Cosmet. Sci.* **2013**, *35*, 238.
